# ﻿*Sideritiscarpetana* (Labiatae), a new high-mountain Mediterranean species from the marble outcrops of the Sierra de Guadarrama (Central System, Madrid, Segovia, Spain)

**DOI:** 10.3897/phytokeys.251.129982

**Published:** 2025-01-17

**Authors:** Jose Luis Izquierdo, Rosina Magaña Ugarte, Alba Gutiérrez-Girón, Concepción Obón de Castro, Diego Rivera Núñez, Rosario G. Gavilán

**Affiliations:** 1 Research and Management Centre of Sierra de Guadarrama National Park, E-28740 Rascafría, Spain Research and Management Centre of Sierra de Guadarrama National Park Rascafría Spain; 2 Botany Unit, Pharmacy Building, University Complutense of Madrid, E-28040 Madrid, Spain University Complutense of Madrid Madrid Spain; 3 Plant Biology Department, University Miguel Hernández of Elche, Avenida de la Universidad s/n, E-03202 Elche, Spain University Miguel Heernández of Elche Elche Spain; 4 Plant Biology Department, University of Murcia, Campus de Espinardo, Edificio nº 20, E-30100 Murcia, Spain University of Murcia Murcia Spain

**Keywords:** Calcareous high-mountain grasslands, Carpetan Mountains, high mountain species, marble outcrops, Mediterranean area, *Sideritissect.Sideritis*, *Sideritissubsect.Fruticulosa*

## Abstract

A new species of *Sideritis* (*Sideritiscarpetana*) is described from the calcareous, high-mountain Spanish flora in the central part of the Iberian Peninsula. It is found in a Mediterranean climate at high-elevation, perennial, calcareous grasslands, as well as in marble screes of anthropogenic origin in the Sierra de Guadarrama, Central System (Spain), in a reserve area within the Sierra de Guadarrama National Park, at 1996 m asl. Taxonomic morphological measurements were performed on collected specimens from Sierra de Guadarrama as well as on geographically-adjacent *Sideritis* (i.e., *S.glacialis*, *S.pungens*, *S.hyssopifolia*). The relationships among them were then explored with multivariate analysis. *Sideritiscarpetana* is a dwarf shrub with an erect or decumbent habit, growing up to 15 cm; non-woody twigs with long hairs of 3–4 cells, leaves are entire, linear-oblanceolate, sparsely covered with trichomes; inflorescence is spiciform or slightly verticillated, flowers are yellow and nutlets ovoid. A key is supplied to help distinguish it from other high-mountain Iberian species included in sectionSideritis. The species is unique in its combination of morphological and autoecological characters. *S.carpetana* shares similarities with *S.glacialis*, a species from Sierra Nevada, and its northern Mediterranean variant, S.glacialissubsp.fontqueriana from Sierra de Gúdar. They share morphological characters that are absent in other high-mountain *Sideritis*, reinforcing their Mediterranean character, as opposed to a more temperate or submediterranean character, such as those of the *hyssopifolia* group.

## ﻿Introduction

The genus *Sideritis* L. contains 140 species found across Eurasia (Russia, Tibet, and western China), with an important centre of diversity in the Iberian Peninsula and Macaronesia. Regarding the Iberian Peninsula, there are 35 species considered in Section Sideritis, and 24 in Macaronesia included in Subgenus Marrubiastrum (Pérez de Paz and Negrín 1990; Las Heras et al. 1999; Ríos et al. 2001; Morales 2016). The number of taxa increases to 100 in one of the most comprehensive works for the genus (Obón and Rivera 1994). *Sideritis* is an interesting genus with a large number of endemics and significant variability in morphological features; but it is further complicated by the hybridization process, which occurs even between species from different sections that coincide in the same area. This variability is also associated with its broad habitat affinity on siliceous or calcareous substrate at all altitudinal levels, while consistently demonstrating a strong heliophilous character (Socorro 1982; Peris et al. 1990; Morales 2016).

On the La Flecha Pass, which connects the provinces of Madrid and Segovia, at an elevation of around 2000 m asl., we discovered a few small populations of *Sideritis* during our fieldwork in the calcareous uplands of Sierra de Guadarrama National Park that, initially appeared to be members of the *Sideritishyssopifolia* group. An in-depth study of the plant revealed similarities with *S.glacialis*, whose closest populations are found in Sierra de Gúdar (Teruel province; ca. 400 km away). Extensive examinations led us to identify *Sideritiscarpetana* as a new species in the Iberian flora, presented in this article.

## ﻿Materials and methods

### ﻿Study area and vegetation

The Central System is a mountain range running northeast-southwestern in the centre of the Iberian Peninsula. It reaches its maximum altitude at 2592 m asl in Sierra de Gredos (Almanzor peak). It divides the “Meseta”, a broad, elevated plateau with an average elevation of 670 m asl, surrounded by other mountains like Montes de León (NW) and the Cantabrian Range (N), which together define the boundary between the Temperate and Mediterranean regions. The Sistema Central comprises the following mountain ranges, from east to west: Sierra de Ayllón, Sierra de Guadarrama, Sierra de Gredos, Sierra de Gata, Peña de Francia and Sierra de Estrela.

**Figure 1.**
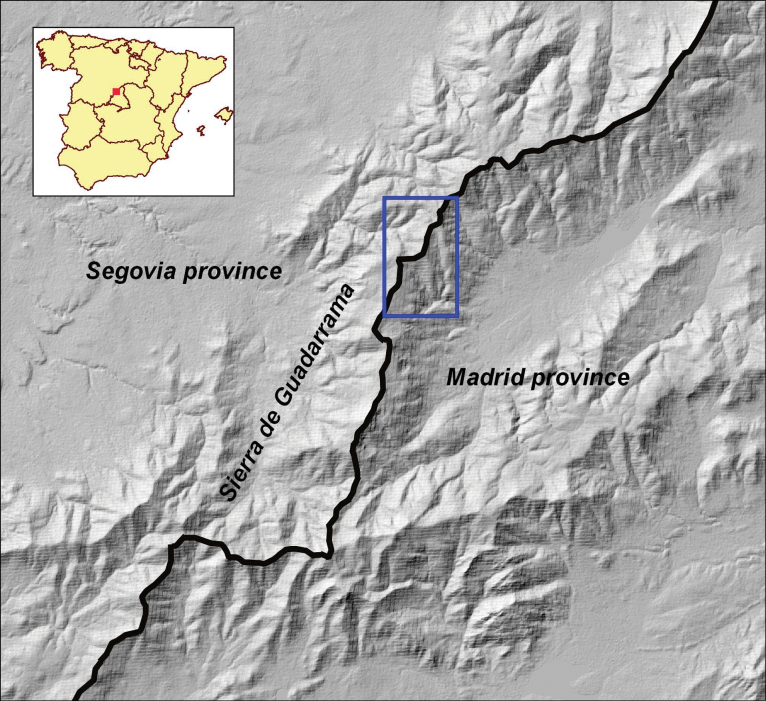
Geographical location of the study region in Spain, between the provinces of Segovia and Madrid. The marble crop status (blue square) is shown on the map. All of these mountains with summits above 1500 m asl are included in the Sierra de Guadarrama National Park.

This new species (*Sideritiscarpetana*) was found in the calcareous peaks of Sierra de Guadarrama. The calcareous outcrops of Sierra de Guadarrama are located in “Collado de la Flecha” (La Flecha Pass) and the head of Artiñuelo creek, within the Sierra de Guadarrama National Park. This area is shared by the provinces of Madrid and Segovia, encompassing the two towns of Rascafría in Madrid (Mountain Public Land n. 153 “Las Calderuelas y otros”) and Trescasas in Segovia (Mountain Public Land n. 257 “La umbría de los Saltillos”). Dolomitic marble outcrops are the predominant feature among the glandular orthogneisses in these mountains. Their distribution is scattered and covers a surface of ca. 120 ha and at an altitude of ranging from 1700 to 2000 m asl (Fig. 1). Prior studies have demonstrated the fascination with this area of the Sierra de Guadarrama, which acts as a genuine “floristic island” amidst the distinctive silicicolous carpetan communities of the Central System. Although there are some notable distinctions, there is also a clear relationship with the closest high mountain limestones, the Gúdar and Javalambre mountains (Southern Iberian System; Pérez Badia et al. 1998; Gavilán et al. 2012).

Floristically, there is a set of taxa found exclusively in this locality within the Sistema Central, such as Astragalusnevadensissubsp.muticus, that grows along with another group of plants that also grow in the foothills of Sierra de Guadarrama (Segovia province) or in the Sierra de Pela (eastern part of Central System), such as *Arenariaerinacea*, *Seselimontanum*, *Iberissaxatilis*, *Teucriumexpassum* or Astragalusincanussubsp.nummularioides. Vegetation is formed by basophilous rocky communities (found in rocky outcrops) interspersed with dry grasslands rich in chamaephytes, dominated by basophilous species with the presence of acidophilous elements.

### ﻿Morphological study

Morphological characters, measurements and detailed descriptions were conducted following the outlined by Rivera and Obón (1990), Obón and Rivera (1994), Rivera et al. (1999), using both fresh materials and dried specimens (preserved herbarium specimens). We have studied high-mountain relatives of *Sideritis* close to our study area to check morphological similarities and differences. The closest areas appear in Sierra de Gúdar and Sierra de Javalambre. We added the Cantabrian Range as the farthest because of the Mediterranean component of its flora that relates it to the others. We did not consider the Pyrenees with a temperate climate, areas more distant the Cantabrian Range nor the Sierra Nevada, a Mediterranean mountain sharing high mountain *Sideritis* species with Sierra de Gúdar (Morales 2016). Populations from Sierra de Guadarrama were compared with specimens of high-mountain *Sideritis* species: from S. de Gúdar and S. de Javalambre, *S.glacialis* (S.glacialissubsp.fontqueriana Obón and Rivera; S. *fernadez-casasii* Rosello et al), *S.pungens* (S.pungenssubsp.javalambrensis) and *Sideritishyssopifolia* (Table 1). Due to the morphological variability in the complex of *S.hyssopifolia*, a comparison was made with the nearest subspecies in terms of distance and altitude, i.e., S.hyssopifoliasubsp.nocedoi Obón and Rivera from the Cantabrian Range (Table 1). The accepted systematic arrangement of *Sideritis* follows Flora iberica (Morales 2016), but takes into account the variability reported in Obón and Rivera (1994). Additional relevant sources studied include Pau (1887); Rivas Goday and Borja (1961); Mateo and López Udías (2000); Mateo and Pisco García (2000); Peris et al. (1994); Roselló et al. (1994) and Roselló et al. (2000). Plant names follow Brummitt and Powell (1992). Acronyms of herbaria are as indicated in Index Herbariorum and its supplement (Holmgren et al. 1990; Holmgren and Holmgren 1993; Thiers (updated continuously)). A key is provided to distinguish all *Sideritis* that can reach high elevations in the Iberian mountains.

**Table 1.** Comparison of *Sideritiscarpetana* sp. nov. with other closely-related taxa. Data on *S.glacialis*, *S.pungens* and *S.hyssopifolia* from Obon and Rivera 1994; Rivera et al. 1999; Roselló et al. 2000; Ríos et al. 2001; Morales 2016 and studied specimens. (See Suppl. material 1).

Characters	*Sideritiscarpetana* sp. nov.	*S.glacialis* (Sierra de Gúdar) = *S.glacialissubsp.fontqueriana = S. fernandez-casasii*	*S.pungens* (Sierra de Javalambre) = S.pungenssubsp.javalambrensis	Sideritishyssopifoliasubsp.nocedoi (Cantabrian Range)
Growth	decumbent to erect	procumbent	erect	procumbent to erect
Height (cm)	10.9–14.7	up to 25	up to 22	up to 45
**Non-woody branches**				
Arrangement of hairs	holotrichous	holotrichous	goniotrichous to holotrichous	goniotrichous to holotrichous
Length of hairs (µm)	700–1280	1000–1500	500–1300	700–1000
N. cells of trichomes	3–4	1–3	2–3	2–3
Trichome cell type	cylindrical	band-shaped	cylindrical	cylindrical
Apical trichome cell type	band-shaped	band-shaped	conical	conical
**Lower leaves**				
Length (mm)	8–12.5	4–9	10–28	7–17
Width (mm)	2.5–3.5	1–2	2–4	2–4
Shape	linear-oblanceolate to oblanceolate	linear-spatulate	linear to oblanceolate	lanceolate-spatulate
Type of apex	acute to apiculate	acute	apiculate	obtuse
Length of trichomes (µm)	700–1260	800–1200	700–1000	200–900
**Inflorescence**				
Length (cm)	2–3	1.5–3	1–6	1–2
Number of verticillasters	5–6	1–7 (8)	(1)4–6	2–5
**Lower verticillaster**				
Direction of bracts	erect-patent to patent	erect-patent	patent	erect-patent
bract shape	ovate or trullate	trullate	ovate	ovate or trullate
bract length (mm)	6.5–9	5–7	8–12	5–8
bract width (mm)	4.8–7.5	6–7	10–12	4–6
Number teeth of semibracts	7–10	9–10 (14)	14–16	0–3
**Abaxial Surface of bracts**				
Gland density	very scarce	absent	scarce to very scarce	absent
Trichome density	scarce to abundant	abundant	scarce to very scarce	scarce to very scarce
Trichome length (µm)	800–1300	800–1200	800–1200	200–800
Apical cell trichome type	cylindrical /band shaped	conical	conical	conical
**Calyx**				
Length in blooming (mm)	6–8.3	5–6 (8)	7–8	5–6
Length teeth	2.5–3.5	1.5–2.5	1–3	1–2
**Corolla**				
length (mm)	8–10	8–10	7–9	5–7

### ﻿Statistical analysis

**Figure 2.**
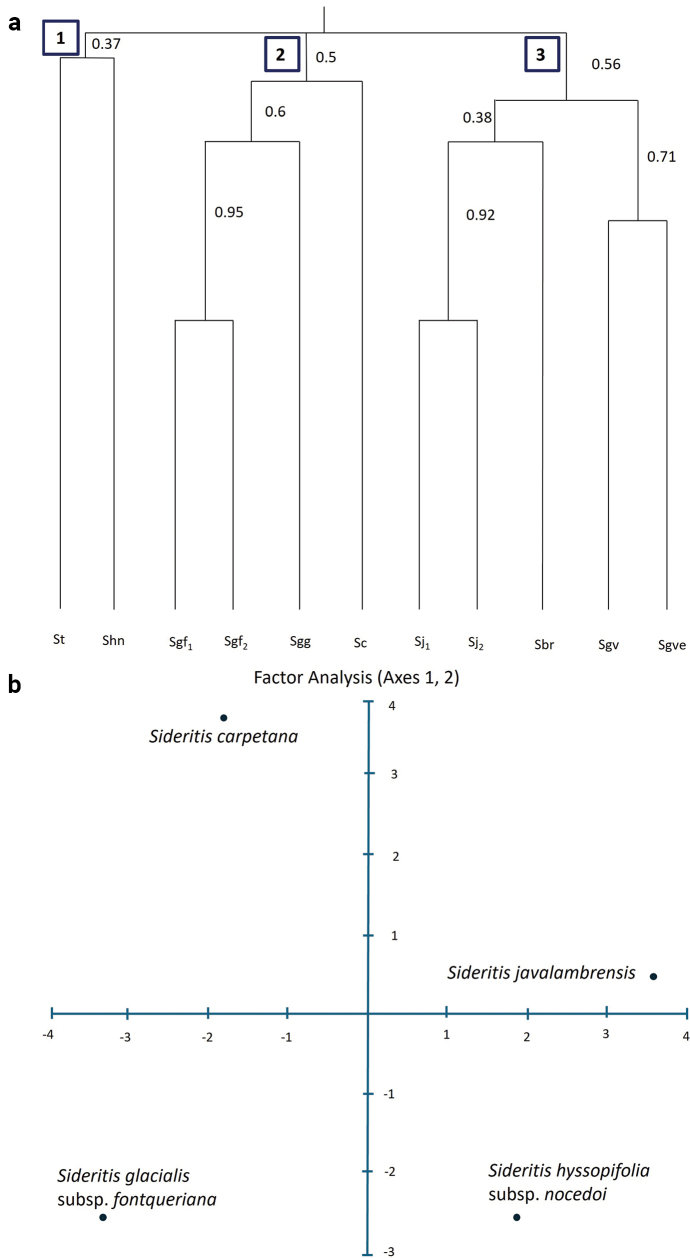
**a** hierarchical clustering (Weighed Neibor Joining) of the Iberian high-mountain Sideritis. The new species *Sideritiscarpetana* is situated in cluster 2 with *Sideritisglacialis* gr. (Sc, *S.carpetana* sp. nov, Sgf, S.glacialissubsp.fontqueriana with two forms 1 for high-mountain areas and 2 for middle areas, Sgg, S.glacialissubsp.glacialis) **b** ordination diagram (Factorial analysis) of *Sideritiscarpetana* and close taxa (Axes 1 and 2). *S.carpetana* is situated close to Sideritisglacialissubsp.fontqueriana along axis 1 but far from the other two. Axis 2 also shows the independence of *S.carpetana* from the other three species.

We conducted an exploratory analysis to understand the relationships between this new species and the other high-mountain Iberian *Sideritis* species. The process was performed in two stages. The first stage consisted of examining all *Sideritis* species present in the Iberian Peninsula at medium to high altitudes that share similarities in morphological characters (Suppl. material 1: table S1). Next, we compared the suspected new species to geographically-close taxa. During the first phase, we differentiated material from Sierra de Gúdar and Javalambre (both for S.glacialissubsp.fontqueriana and *S.javalambrensis*) into two forms based on altitude range: Form 1 for specimens from the highest altitude, and Form 2 for those from lower altitudes (see Fig. 2). Doing so allowed for more accurate definitions on the altitudinal criteria of the species under study. We performed an agglomerative clustering method by means of Weighted Neighbor Joining, using the Sokal-Sneath index of dissimilarity (un2) supported by 500 bootstraps for 89 morphological variables of high-mountain Iberian *Sideritis* (Rivera et al. 2014; Röhrs et al. 2023). In the second stage, we conducted an ordination method using Factor Analysis, a model of the measurement of a latent variable that cannot be directly measured with a single variable. This is a reduction technique that captures the variance in a small set of variables (Howard 2016; Tavakol and Wetzel 2020). We implemented the same dissimilarity index as in the clustering analysis.

## ﻿Results

The multivariate analysis results indicated that this species is sufficiently independent from the rest of *Sideritis* dataset (Fig. 2a, b). The dendrogram of the hierarchical clustering (Fig. 2a) displays three main groups. The first group of the dendrogram (1, Fig. 2a) includes S.hyssopifoliasubsp.nocedoi from the Cantabrian Range (Shn; Fig. 2a) and *S.tugiensis* from southeastern mountains of the Iberian Peninsula, Sierra de Segura (St; Fig. 2a). The second group (2, Fig. 2a) includes the new *Sideritis* from Sierra de Guadarrama (Sc; Fig. 2a) and a group of species included in *S.glacialis*. The specimens from the summits of Sierra Nevada, in the south of the Iberian Peninsula (S.glacialissubsp.glacialis, Sgg; Fig. 2a), and the two forms from Sierra de Gúdar in central eastern Spain (S.glacialissubsp.fontqueriana, Sgf1, Sgf2; Fig. 2a), which show strong similarity, suggesting they could be treated as belonging to the same subsection. A third group (3, Fig. 2a) consists of two smaller subgroups. One subgroup is from Sierra Nevada and related to *S.glacialis* but from lower altitudes (oromediterranean belt) and ascribed to S.glacialissubsp.vestita (Sgve; Fig. 2a) and S.glacialissubsp.virens (Sgv; Fig. 2a). The second subgroup comprises those *Sideritis* species found in Sierra de Javalambre in the central eastern part of the Iberian Peninsula (*S.pungens* or *S.javalambrensis*, Sj1, Sj2; Fig. 2a).

Factor analysis ordination showed the separation of the new *Sideritis* species from closely related ones along axis 1 and axis 2 (Fig. 2b). The first factor analysis axis shows how Guadarrama *Sideritis* and *S.glacialis* from Sierra de Gúdar (*S.g.fontqueriana*) are separated in the negative part; while *S.h.nocedoi* from Cantabrian Range and *S.javalambrensis* (= *S.pungens*) from Sierra de Javalambre are separated in the positive part. The second axis clearly distinguishes the Guadarrama *Sideritis* from *S.javalambrensis* in the most positive part of the axis, which is also in the positive part but closer to 0; and separates *S.glacialis* from Sierra de Gúdar and *S.nocedoi* from Cantabrian Range in the negative part.

The results from the multivariate analyses clearly recognize the independence of this new *Sideritis* species from Sierra de Guadarrama. Additionally, the morphological analyses results (Table 1, Appendix 1) led to the identification of a new species from the summits of Sierra de Guadarrama that we are going to call *Sideritiscarpetana* Izquierdo & Gavilán, sp. nov.; that is to be included under Sect. Sideritis, subsect. Fruticulosa due to its relationships with Sideritisglacialissubsp.fontqueriana from Sierra de Gúdar, as shown in Fig. 2a, b. The following text presents the diagnosis, holotype and other characteristics of this new species, including phenology, distribution, habitat and the detailed description with all morphological characters studied.

### ﻿ Sideritis carpetana

Taxon classificationPlantaeLamialesLabiatae

Izquierdo & Gavilán sp. nov

6B3EC8C6-6BBF-59FF-9547-DF257E8E3330

urn:lsid:ipni.org:names:77355303-1

#### Description

**Figure 3.**
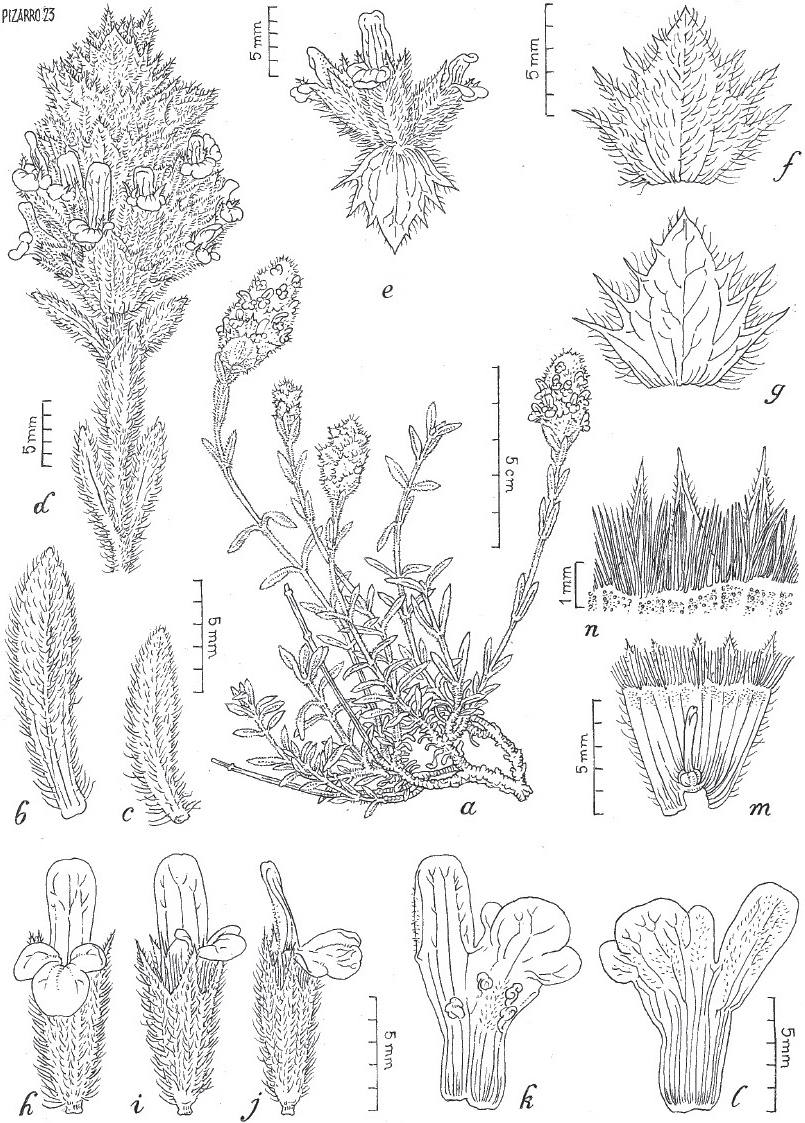
*Sideritiscarpetana*, Sierra de Guadarrama, La Flecha Pass (1996 m asl) **a** habit **b, c** leaves **d**, inflorescence detail **e** verticillaster **f, g** bracts, lower face (**f**) upper face (**g**) **h, i, j** flower, front and lateral view **k, l** corolla detail showing the inner part with stamens (**k**) and outer part (**l**) **m, n** open calyx with ginoecious (**m**) and teeth detail (**n**). Drawing by J. Pizarro.

**Figure 4.**
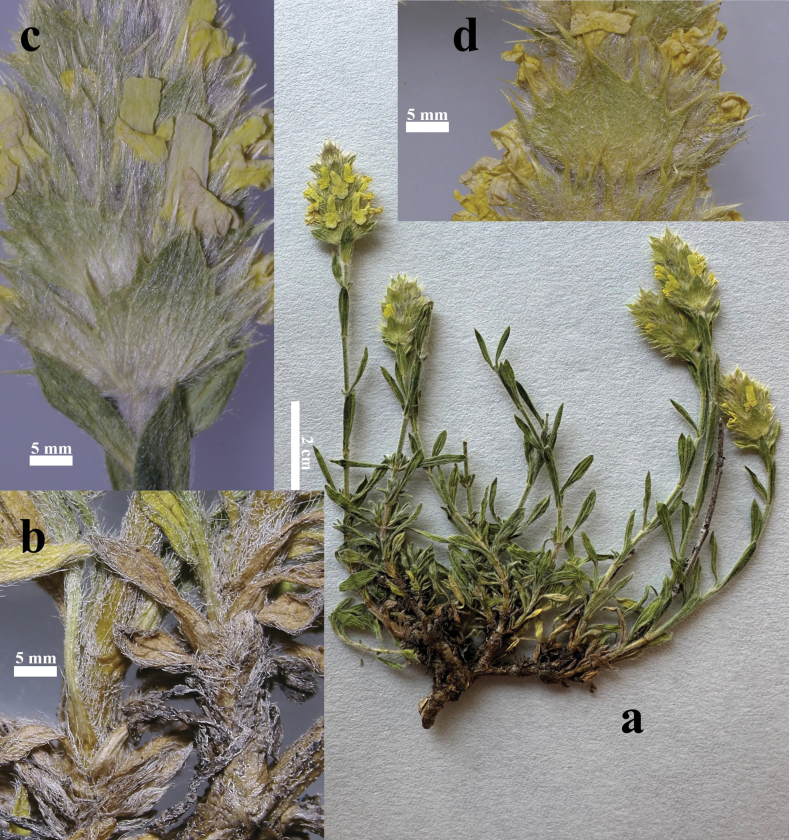
*Sideritiscarpetana*, Sierra de Guadarrama, La Flecha Pass (1996 m asl) **a** habit **b** Lower branches **c** inflorescence detail showing lower verticillaster bract and calyx **d** inflorescence detail showing upper verticillaster bract.

**Figure 5.**
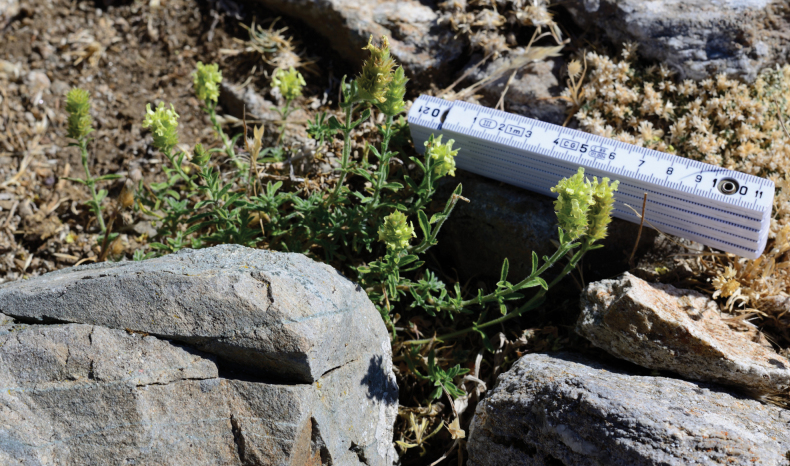
*Sideritiscarpetana* natural habit in the marble anthropogenic outcrops of La Flecha Pass (Sierra de Guadarrama National Park).

**(Figs 2–5).** Dwarf shrub with erect or decumbent habit, growing up to 25 cm. Stems are holotrichous with white hairs. Branchlets at the base have cylindric trichomes of 3–4 cells, except the apical band-shaped being 0.7–1.3 mm long. Leaves are entire, linear-oblanceolate, measuring 8–12.5 × 2.5–3.5 mm long, markedly trinerved and mucronated, sparsely covered by trichomes on the upper side of leaf and more densely covered on the underside. Middle bracts measure 6–8 mm. The inflorescence is spiciform or slightly verticillated with 5–6 whorls, each enclosing 5(6) flowers. The calyx is campanulate, densely haired, 6–8.3 mm long, teeth spiny-acuminate, equal, and erect, 2.5–3.5 long, with continuous carpostegium, spines 1–1.5 mm. The corolla is yellow 8–9 (10) mm, with entire or two slight lobes in the upper lip and three in the lower lip, the central bigger than the other two; stamens included in the corolla tube, with filaments 4–5 mm long; style 3–3.5 mm long; ovoid nutlets 1 mm (see Suppl. material 1: table S1 for diagnostic features and detailed description).

This species lives in the mountains of Sierra de Guadarrama, in the marble outcrop reserve of La Flecha Pass, in the Sierra de Guadarrama National Park, on Mediterranean high-mountain calcareous grasslands and screes of anthropogenic origin, mainly in southern exposures, at an altitude of 1990–2000 m asl.

#### Holotype.

• Holotype: Spain, Madrid: Sierra de Guadarrama. Collado de la Flecha, 1996 m asl. 14/07/2021. 40.928276, -3.925597 (ETRS89), *Rosario G. Gavilán* and *José Luis Izquierdo*, MAF [MAF 181439].

#### Phenology.

The species is known to have its prime flowering season from June to July; and it is reported to fruit from August to October.

#### Distribution and habitat.

*Sideritiscarpetana* grows in Mediterranean high-mountain calcareous grasslands of *Festuco-Ononidetea striatae* (*Festuco carpetanae-Astragaletum mutici*) and in marble screes of anthropogenic origin at La Flecha Pass, Sierra de Guadarrama, at an altitude of 1996 m asl (Table 2). The geographical distribution is restricted to the marble outcrop reserve within the Sierra de Guadarrama National Park. In these territories, winter snow cover lasts for approximately five months, while summer rainfall is scarce as is typical of a Mediterranean territory.

**Table 2.** Phytosociological relevés recorded in La Flecha Pass, Association *Festucocarpetanae-Astragaletummutici* Gavilán, Díez-Monsalve, Izquierdo, Gutiérrez- Girón, Fernández-González & Sánchez-Mata (*Sideritido-Arenarion*, *Festucohystricis-Poetalialigulatae*, *Festucohystricis-Ononideteastriatae*). Species Indices and phytosociological association typology (vegetation type) follow J. Braun-Blanquet (1928).

Altitude	1996	2000	1995	1995	1990	1995	1990	1995
Area	4	1	2	1	1.5	1.5	1	2
Exposure (°)	182	350	160	176	203	189	117	150
Plot number	1	2	3	4	5	6	7	8
*Festuco-Ononidetea* characteristics								
*Sideritiscarpetana*	4	3	2	3	3	3	2	3
*Poaligulata*	1	1	1	1	1	+	2	2
Astragalusnevadensissubsp.muticus	3	.	1	.	.	1	+	.
*Seselimontanum*	.	1	+	+	1	+	1	1
*Iberissaxatilis*	2	.	.	+	.	2	.	.
*Arenariaerinacea*	.	.	.	.	+	.	.	.
*Bupleurumranunculoides*	1	.	.	.	.	.	.	.
*Veronicajavalambrensis*	+	.	.	.	.	.	.	.
*Festuceteaindigestaedifferentials*								
*Festucacurvifolia*	1	2	.	1	1	.	2	2
*Leucanthemopsispallida*	.	+	1	1	2	1	1	.
*Thymuspraecox*	.	+	+	+	+	.	.	.
*Jurineahumilis*	.	+	.	.	.	+	.	.
Dianthuspungenssubsp.brachyanthus	.	.	.	+	.	.	.	.
Other species								
Arenariaobtusiflorasubsp.ciliaris	+	.	+	+	+	+	1	1
*Centaureatriumphetii*	+	.	1	1	.	.	+	.
*Cynosurusechinatus*	2	.	+	.	+	+	.	.
Coronillaminimasubsp.minima	.	.	+	+	1	.	.	.
*Festucaiberica*	+	.	+	.	+	.	.	.
*Agrostiscastellana*	+	+	.	.	.	.	.	.
*Alliumsphaerocephalon*	.	.	+	+	.	.	.	.
*Carduuscarpetanus*	+	.	1	.	.	.	.	.
*Bromustectorum*	.	.	.	.	.	.	.	+
*Acinosalpinos*	.	.	.	.	.	+	.	.
*Ceterachofficinarum*	.	.	.	.	.	.	.	.
*Cetrariaaculeata*	.	+	.	.	.	.	.	.
*Galiumidubedae*	.	+	.	.	.	.	.	.
*Chaenorrhinumsegoviense*	1	.	.	.	.	.	.	.
*Herniariacinerea*	.	1	.	.	.	.	.	.
*Holcusgayanus*	+	.	.	+	+	+	+	+
*Hornungiapetraea*	+	.	.	.	.	.	.	.
*Lactucaviminea*	.	.	.	.	.	+	.	.
*Nardusstricta*	.	1	.	.	.	.	.	.
*Poabulbosa*	.	.	.	.	.	+	+	+
*Rumexangiocarpus*	+	.	.	.	.	.	.	.
*Saxifragagranulata*	.	.	.	.	.	.	.	.
*Sedumalbum*	+	1	.	+	1	1	+	+
*Silenelegionensis*	+	.	1	.	.	+	.	.
*Teesdalianudicaulis*	.	.	+	.	.	.	.	.
*Thlaspistenopterum*	.	.	.	.	+	.	+	+
*Violariviniana*	.	.	.	2	1	.	.	.

#### Etymology.

The specific epithet ‘carpetana’ refers to the former inhabitants of these mountains that were called ‘carpetanos’ (carpetan, in English). Moreover, “Cordillera Carpetana”, or Carpetan Range, is a synonym for Sistema Central (Central System), the mountain range that divides the northern and southern plateau in central Iberian Peninsula. The Sistema Central comprises several mountain ranges: Sierra de Estrela, Sierra de Gata, Peña de Francia, Sierra de Gredos, Sierra de Guadarrama and Sierra de Ayllón from western to eastern territories.

### ﻿Key to the Iberian high mountain Sideritis (> 1996 m asl)

**Table:** 

1	Plant with some petiolated leaves	***S.lurida* J. Gay ex Lacaita**
–	Leaves without petiole	**2**
2	Leaves linear or lanceolate, entires	**3**
–	Leaves lanceolate, elliptic, ovate u obovate, toothy o lobed	**4**
3	Inflorescence < 1.5 cm	***S.carbonellii* Socorro**
–	Inflorescence > 2 cm	**5**
4	Leaves less than 3 mm wide	**6**
–	Leaves bigger than 3 mm wide	**7**
5	Bracts < 6 mm	***S.incana* L.**
–	Bracts > 6 mm	***S.pungens* Benth.**
6	Hairs of the shoot base short (< 1 mm)	**8**
–	Hairs of the shoot base long (> 1 mm)	**9**
7	Verticillated inflorescence	***S.hirsuta* L.**
–	Spiciform or slightly verticillate inflorescence	**9**
8	Trichomes of base branchlets goniotrichous to holotrichous, with 1–3 cells, the apical conic.	**S.gr.hyssopifolia L.**
–	Trichomes of base branchlets holotrichous, with 3–4 cells, the apicalband-shaped.	**9**
9	Hairs of the shoot base 1–2.5 mm	**S.gr.glacialis Boiss.**
–	Hairs of the shoot base 0.7–1.3 mm	***S.carpetana* spec. nov.**

## ﻿Discussion

Plants living on the marble outcrops of Sierra de Guadarrama enrich the prevalent silicicolous flora of the area. The majority of these calcareous plants are found in foothill areas, but a small number of them reach altitudes above the timberline (1800–2000 m asl) and are thus included in the Sierra de Guadarrama National Park reserve. These outcrops have been identified as a floristic island surrounded by the characteristic glandular orthogneisses of these mountains (Pérez Badia et al. 1998; Gavilán et al. 2012).

According to Obón and Rivera (1994) and Rivera et al. (1999), section Sideritis, subsection Fruticulosae includes *Sideritis* with long hairs covering the base of the branchlets (Obon and Rivera 1994); it is also holotrichous, formed by cylindrical cells, except the apical band-shaped (forming bands along the cell). This subsection consists of five species (*S.fruticulosa*, *S.spinulosa*, *S.subspinosa*, *S.jahandiezii* and *S.glacialis*), but the only species growing at high altitudes in the cryoromediterranean belt are those of the *S.glacialis* complex (subsp. glacialis and subsp. fontqueriana) or oromediterranean (subsp. vestita and subsp. virens). These species inhabit the south of the Iberian Peninsula (Sierra Nevada and surroundings mountains), with the exception of subsp. fontqueriana, which grows at the Sierra de Gúdar which is close to Sierra de Guadarrama, and whose specimens have been studied in the comparison with *Sideritiscarpetana*.

Sideritis belonging to subsection Hyssopifoliae have shorter hairs at the base of the branchlets, goniotrichous to holotrichous, formed by cylindrical cells, and the apical cell is conical. This subsection includes 7 species (*S.brachycalix*, *S.getula*, *S.hyssopifolia*, *S.pungens*, *S.maura*, *S.carbonellii* and *S.ochroleuca*; Obon and Rivera 1994). The species found in high mountain areas are *S.carbonellii*, *S.pungens* and *S.hyssopifolia*. We have compared with *Sideritiscarpetana* those found in locations close to Sierra de Guadarrama and on calcareous substrate: i.e., *S.pungens* (subsp. javalambrensis, Sierra de Javalambre) and *S.hyssopifolia* (subsp. nocedoi, Cantabrian Range). After comparing the studied characters of all the selected species in both subsections (see Table 1), we included *S.carpetana* in subsection Fruticulosae. This is due to the fact that it has long hairs at the base of the branchlets, which are formed by 3–4 cells (including the apical, that is band-shaped) while in the others these are conical. Like all species in this subsection, hairs in the inflorescence axis or bracts are similar to those at the base of branchlets. Morphological findings have been validated by multivariate analyses.

Similarity in macromorphological characters with other orophilous *Sideritis*, such as hair length and the type of leaves, demonstrates the ecological convergence of these species due to the harsh environmental conditions in high mountain areas and the intense speciation process (Ríos et al. 2001). This is the case for the *S.glacialis* complex, which includes four mountainous taxa, and *S.hyssopifolia*, with two subspecies with an orophilous distribution, subsp. nocedoi from the Cantabrian Range and subsp. eynensis from eastern Pyrenees. Although Sierra de Guadarrama is sometimes thought of as submediterranean due to a slightly shorter summer arid season compared to southern territories like Sierra Nevada, it shares similarities in ecology, distribution, and characters with the Sierra de Gúdar Sideritis (*S.glacialis*, S.glacialissubsp.fontqueriana). This is evident in *S.carpetana* which has long trichomes at the base of non-woody twigs and other parts, as well as the holotrichous indumentum, which is similar to taxa found in Mediterranean zones. This marked Mediterranean character of Sierra de Guadarrama and its relationships with Sierra Nevada vegetation has been highlighted in recent revisions of high Iberian vegetation (Gavilán et al. 2017). On the other hand, the rhizomatous habit is also a typical characteristic of orophilous taxa, for instance *S.lurida* (subsect. Lurida), found close to the Sierra de Gredos (Central System), grows in loose substrates (screes) similar to *S.carpetana*, although these are of geological origin and not anthropogenic, as in our case (Rivera and Obón 1990; Luceño et al. 2000).

A comprehensive flora checklist for the siliceous substrate, which is more prevalent along the Central System exists (Rivas-Martínez 1963; Sánchez-Mata 1988, Fernández-González 1988; Luceño et al. 2000), while the scarce and dispersed calcareous areas, which have a particular flora, have received less attention. However, there have been recent discoveries of new plant species in those well-known areas, such as the Sierra de Guadarrama (Fernández-González and Izco 1989). In the uplands of La Flecha Pass, the marble outcrops are interspersed with the typical preponderance of orthogneisses that conceal the calcareous landscape. Their preservation is currently a top priority in the Sierra de Guadarrama National Park. It has applied conservation measures to this natural reserve, as outlined in the Spanish National Parks Law 30/2014, which include several uses that are incompatible with the current management practices, such as public usage (visits) and substantial animal grazing (livestock). The area is currently described as having high ecological significance and unique natural assets that are of exceptional rarity, fragility, and scientific interest in order to ensure proper protection for these marble outcrops.

## Supplementary Material

XML Treatment for Sideritis carpetana

## ﻿Additional information

### Author ORCIDs

Rosina Magaña Ugarte https://orcid.org/0000-0003-0628-3251

Alba Gutiérrez-Girón https://orcid.org/0000-0002-0988-3343

Concepción Obón de Castro https://orcid.org/0000-0002-0244-601X

Diego Rivera Núñez https://orcid.org/0000-0001-6889-714X

Rosario G. Gavilán https://orcid.org/0000-0002-1022-445X

### Data availability

All of the data that support the findings of this study are available in the main text or Supplementary Information.

## ﻿Appendix 1

Specimens studied.

***Sideritisglacialis* (incl. subsp. fontqueriana , *S.fernandez-casasii*)**

TERUEL: Puerto de Valdelinares, 30TYX07, 1800 m asl, in Junipero sabinae-Pinetum sylvestris. *Andrés Molina*, 5-8-1982, MAF 120116; Altos de la Sierra de Gúdar. *Salvador Rivas Goday*, 24-6-1946, MAF 77086; 4-7-1995, Valdelinares, Sierra de Gúdar, estación invernal, *Vicente Jesús Arán*, MA561273; Valdelinares, próximo a la desviación de Peñaroya, 29-08-2002, *J. Herrero*, MA700968; Cerca de la Virgen de la Vega, Alcalá de la Selva, Collado de la Gitana, 10-7-1946, *Font Quer, Sierra y Torres*, MA344902; Sierra de Gúdar, 1900 m, 5-8-1974, *G. López & E. Valdés-Bermejo*, MA435326.

GRANADA: Sierra Nevada, 24-8-1977, *E. Rico*, MA 212210; Capileira, Sierra Nevada, Vasares del Veleta, laderas rocosas y pedregosas, micaesquistos, 3020 m asl, 19-8-1990, *L.F. Sánchez & J.A. Alejandre*, MA 493728

***Sideritishyssopifolia* (incl. subsp. nocedoi)**

LEÓN: Boca de Huérgano, macizo de Peña Prieta, roquedos calizos en laders de solana del valle de Lechada, 1970-2060 m asl, 16-7-1990, *M.L. Gil Zúñiga & J.A. Alejandre*, MA493729; San Emiliano, Peña Ubiña, SE, laderas pedregosas calcáreas, 30TTN5967, 2000 m, 30-7-2001, *V.J. Arán & M.J. Tohá*, MA691953; Circo Cebollero, Puerto de San Isidro, 27-7-1979, *Casaseca, Fernández, Amich, Rico & Sánchez*, MA256354; Between Nocedo and Valdeteja, 1800 masl, 27-8-1972, *Salvador Rivas Goday and Jesús Izco*. MA256357, *ibidem* MAF84289.

***Sideritispungens* (incl. subsp. javalambrensis , *S.javalambrensis*)**

TERUEL: Camarena de la Sierra, Javalambre peak, 30TXK6841, 2000 m asl, in lapidosis calcareis, 18-9-1981, leg. *Santiago Castroviejo*, *Fernández Quirós*, det. *José Borja*. MAF 127937; *ibidem* MA381171; La Puebla de Valverde, cerro de Javalambre, junto a las instalaciones de televisión, 7-7-1995, *Vicente Jesús Arán*, MA561283; Sierra de Javalambre, 2000 m, 11-9-1900, *F. Sennen*, MA100605; Pico Javalambre, matorrales pulvinulares de Erodio-Erinacetum, 1-7-1983, *Peris, Stubing & González*, MA479758.

## Supplementary materials

Supplementary material 1 Supplementary information Data type pdf Explanation note Detailed description on *Sideritiscarpetana*. **table S1.** Data on taxa included in the multivariate analysis done to the Iberian Sideritis from high and medium altitudes. **table S2.** Comparison of *Sideritiscarpetana* sp. nov. with other closely-related taxa. https://binary.pensoft.net/file/1222110This dataset is made available under the Open Database License (http://opendatacommons.org/licenses/odbl/1.0/). The Open Database License (ODbL) is a license agreement intended to allow users to freely share, modify, and use this Dataset while maintaining this same freedom for others, provided that the original source and author(s) are credited.Jose Luis Izquierdo, Rosina Magaña Ugarte, Alba Gutiérrez-Girón, Concepción Obón de Castro, Diego Rivera Núñez , Rosario G. Gavilán
