# Supplementary material for: ﻿Sideritiscarpetana (Labiatae), a new high-mountain Mediterranean species from the marble outcrops of the Sierra de Guadarrama (Central System, Madrid, Segovia, Spain)
Source: PhytoKeys. 2025 Jan 17;251:143–59. doi: 10.3897/phytokeys.251.129982 (PMC11759922; doi:10.3897/phytokeys.251.129982)
Supplement: Supplementary material 1 — Supplementary information [file phytokeys-251-143_article-129982__-s001.pdf]

Supplementary material

Detailed description

We have followed Obon de Castro and Rivera (1994) features.

Basal parts. Habit, rhizomatous, erect or decumbent; height, 10.9–14.7 cm (13.73). Non woody twigs, erect to little ascendent, 7–13 cm long; indumentum holotrichous, homotrichous; sessile? glands, scarce; glandular hairs absent; trichomes scarce to abundant, antrorse, 700–1280 µ long (872,57±31,86); cells 3–4, cylindrical except the apical band-shaped. **Lower leaves**, 8–12.5 mm long, 2.5–3.5 mm wide, epetiolate, entire, linear-ovolanceolate to ovolanceolate, apex acute to apiculate; sessile glands absent or very scarce glandular hairs absent trichomes scarce;, indistinct, 700–1260 µm long (871,2±31,54); cells, 2–3, cylindrical except the apical band shaped?. **Upper leaves**, similar to the lower. **Axillary fascicles** frequent. **Branches** without side shoots. **Inflorescence** short, ovoid, dense with attenuated apex, 2–3 cm long with colour similar to the vegetative part; with 5–6 verticillasters; sessile glands scarce; glandular hairs absent; trichomes, abundant, antrorse, 840–1420 µm (1100,8±33,76); cells 3–4, cylindrical, the apical cylindrical or band shaped (50%). **Bracts of lower verticillaster**, erect-patent to patent, narrowly ovate, 6.5–9 mm long 4.8–7.5 mm; relative position of the greatest width verticillaster, basal to sub-basal; basal emargination deep and wide; division, shallow; number of teeth of the semibracts, 7–10. **Bracts of middle verticillaster**, erect-patent to patent, ovate, 6–8 mm long, 6–9.5 mm width; relative position of the greatest width verticillaster, central; basal emargination, deep and wide; division, shallow to deep; number of teeth of the semibracts, 6–7; adaxial surface glabrous; abaxial surface very scarce in glands, without glandular hairs; abaxial surface trichomes scarce to abundant; 800–1300 µm long (1052,8±32,64); number of cells, 3–4, cylindrical, the apical cilindrical-band shaped (50%). **Flowers** 6–8 per verticillaster. **Bracteoles** absent. **Calyces**, campanulate in fruit, 6–8.3 mm long when flowering; teeth, 2.5–3.5 mm long; calyx spinules, 1–1.5 mm; carpostegium continuous; sessile glands on external surface abundant; glandular hairs absent; trichomes abundant, 760–1420 µm long (1140±35.06). **Corolla** yellow, 8–10 mm; lips divided, 1/2–1/4of corolla length; filaments 4–5 mm long; style 3–3.5 mm long; mature nuts 1 mm .

Table S1. Data on taxa included in the multivariate analysis done to the Iberian Sideritis from high and medium altitudes. Between brackets the acronims used in Fig. 2 (dendrogram): Sc, *S. carpetana* sp. nov.; Sgg, *S. glacialis* subsp. *glacialis*; Sgf1 and Sgf2, *S. glacialis* subsp. *fontqueriana* from high-mountain areas and from middle areas, respectively; Sj1 and Sj2, *S. pungens* subsp. *javalambrensis* from high-mountain areas and from middle areas, respectively; Shn, *S. hyssopifolia* subsp. *nocedoi*; Sbr, *S. borgiae* subsp. *relegata*; Sgve, *S. glacialis* subsp. *vestita*; Sgv, *S. glacialis* subsp. *virens*; St, *Sideritis tugiensis*.

Taxa	Sideritis carpetana sp. nov. (Sc)	S. glacialis subsp. glacialis (Sgg)	S. glacialis subsp. fontqueriana (Sgf1)	S. glacialis subsp. fontqueriana (Sgf2)	S. pungens subsp. javalambrensis (Sj1)	S. pungens subsp. javalambrensis (Sj2)	Sideritis hyssopifolia subsp. nocedoi (Shn)	S. borgiae subsp. relegata (Sbr)	S. glacialis subsp. vestita (Sgve)	S. glacialis subsp. virens (Sgv)	Sideritis tugiensis (St)
Central verticillaster Adaxial surface hair covering abundant	0	0	0	1	0	1	0	0	0	0	0
Central verticillaster Adaxial surface hair covering glabrous	1	0	1	0	1	0	1	1	0	0	1
Central verticillaster Adaxial surface hair covering scarce	0	0	1	1	1	0	0	1	0	0	0
Central verticillaster bract length 4-6 (mm)	0	1	1	1	1	1	1	0	1	0	0
Central verticillaster bract length 6-8 (mm)	1	0	0	0	1	1	1	0	1	1	0
Central verticillaster bract width 10-15 (mm)	0	0	0	0	1	1	1	0	0	0	1
Central verticillaster bract width 5-10 (mm)	1	1	0	0	1	1	1	1	1	1	1
Central verticillaster semibracts teeth number 0-5	0	0	1	1	1	1	0	1	0	0	1
Central verticillaster semibracts teeth number 10-15	0	1	0	1	0	0	0	0	0	0	0
Central verticillaster semibracts teeth number 5-10	1	1	1	0	1	1	1	0	1	0	1
Lower verticillaster bract length 4-8 (mm)	1	1	1	0	1	1	1	1	1	1	1
Lower verticillaster bract length 8-12 (mm)	1	1	0	0	0	0	1	0	1	0	0
Lower verticillaster bract shape ovate	1	1	0	0	1	1	1	1	1	1	1
Lower verticillaster bract shape trullate	0	0	1	1	0	0	1	0	0	0	0
Lower verticillaster bract width 4-8 (mm)	1	1	1	1	1	1	1	0	0	0	1
Lower verticillaster bract width 8-12 (mm)	0	1	1	1	0	0	1	0	0	0	0
Lower verticillaster Direction of bracts erect-patent	1	1	1	1	0	0	1	0	0	0	1
Lower verticillaster Direction of bracts patent	1	1	0	0	1	1	0	1	0	1	0
Lower verticillaster Direction of bracts reclinate	0	1	0	0	0	0	0	0	0	0	0
Number flowers verticillaster 1-5	0	0	1	0	0	0	1	0	0	0	0
Number flowers verticillaster 5-10	1	1	1	1	1	0	1	1	1	1	1
Apical trichome cell type band shaped	1	0	0	0	1	1	1	0	1	1	1

Apical trichome cell type conical	0	1	0	0	1	1	1	1	1	0	1
Arrangement of hairs goniotrichous WB	0	1	1	1	0	0	0	0	1	0	0
Arrangement of hairs holotrichous WB	1	1	1	1	1	1	1	1	1	1	1
Indumentum heterotrichous WB	0	0	0	0	1	1	0	1	1	1	1
Indumentum homotrichous WB	1	1	1	1	0	0	1	0	1	0	0
Length of hairs 1300-1700 (µm)	0	0	0	0	1	1	0	0	1	1	0
Length of hairs 500-900 (µm)	1	0	0	0	1	1	1	1	0	0	1
Length of hairs 900-1300 (µm)	1	1	1	1	1	1	0	1	1	1	1
N. cells of trichomes 1-2	0	0	0	1	0	1	1	0	0	0	0
N. cells of trichomes 3-4	1	1	1	1	1	1	1	1	1	1	1
Trichome cell type band-shaped	0	0	0	0	0	0	1	0	0	0	1
Trichome cell type cylindrical	1	0	1	1	1	1	1	1	1	1	0
Trichomes antrorse	1	1	1	1	1	1	1	1	1	1	1
Trichomes patent or retrorse	0	0	0	0	0	0	0	1	0	0	0
Lower leaves Density of trichomes abundant	0	1	1	1	0	0	0	0	0	0	0
Lower leaves Density of trichomes scarce	1	1	1	1	1	1	1	1	1	1	1
Lower leaves Density of trichomes very scarce	0	1	1	1	0	0	0	0	1	1	0
Lower leaves Length 12-16 (mm)	0	1	1	1	1	0	0	0	0	1	1
Lower leaves Length 16-35 (mm)	0	0	0	0	1	1	0	0	1	1	1
Lower leaves Length 4-8 (mm)	0	0	0	1	1	0	0	1	1	1	0
Lower leaves Length 8-12 (mm)	1	1	1	1	0	1	1	0	1	1	1
Lower leaves Length of trichomes 1000-1400 (µm)	1	0	1	1	1	0	0	1	0	1	0
Lower leaves Length of trichomes 1400-1800 (µm)	1	1	0	1	1	0	0	1	1	0	0
Lower leaves Length of trichomes 1800-2200 (µm)	0	1	0	1	1	0	0	1	1	0	0
Lower leaves Length of trichomes 200-600 (µm)	0	0	0	1	0	1	0	0	0	0	0
Lower leaves Length of trichomes 600-1000 (µm)	1	0	1	1	1	1	1	0	0	0	0
Lower leaves Type of apex acute	0	1	0	0	0	0	1	1	1	1	0
Lower leaves Type of apex apiculate	0	0	0	0	1	1	0	1	1	1	0
Lower leaves Type of apex obtuse	0	0	0	0	0	0	1	1	0	1	0

Lower leavesWidth 1-2 (mm)	0	0	0	0	1	1	1	1	0	1	1
Lower leavesWidth 2-4 (mm)	1	1	1	0	1	0	1	0	1	1	1
Lower leavesWidth 5-10 (mm)	0	0	0	0	0	0	0	1	0	0	0
Upper leaves different	0	0	0	0	1	1	0	1	1	1	0
Upper leaves Similar to lower leaves	1	1	1	1	1	1	1	0	1	1	0
Apical cell type trichome axis band shaped	1	1	1	0	0	0	0	0	0	0	1
Apical cell type trichome axis conical	1	1	1	1	1	0	1	1	1	1	1
Apical cell type trichome axis cylindrical	0	0	0	0	0	0	0	0	1	0	0
Density glandular hair axis absent	1	0	1	1	1	1	1	1	0	0	0
Density glandular hair axis very scarce	0	1	0	0	1	1	1	1	0	0	0
Distance middle verticillasters dense	1	1	1	1	1	1	1	0	1	1	1
Distance middle verticillasters medium	0	0	0	1	0	1	1	0	1	0	0
Inflorescence Length 1-3 (cm)	1	1	1	1	1	1	1	1	1	1	1
Inflorescence Length 3-6 (cm)	0	1	0	0	0	1	0	1	1	1	0
Number cells trichome axis 1-2	0	0	0	0	0	0	1	1	0	1	1
Number cells trichome axis 3-4	1	0	1	1	1	1	1	1	1	1	1
Number of verticillasters 1-4	0	0	0	1	1	1	1	1	1	1	0
Number of verticillasters 4-8	1	0	0	0	1	1	1	0	1	1	1
Cushion shaped	0	0	0	0	1	1	1	1	0	0	0
Decumbent to erect	0	1	1	1	0	1	1	0	1	1	1
Erhizomatous	1	0	0	1	1	1	1	0	1	1	1
Height 10-15 cm	1	1	1	1	0	0	1	1	1	1	1
Height 15-25 cm	0	0	0	1	0	1	1	1	1	0	0
Height 25-45 cm	0	0	0	0	0	0	1	1	0	1	0
Rhizomatous	0	0	1	1	0	0	0	0	0	0	0
Corolla upper lip apex emarginate	0	1	1	0	1	1	1	1	1	1	1
Corolla upper lip apex entire	1	0	0	0	0	0	0	1	0	0	0
Corolla upper lip apex notched	0	0	0	0	0	0	1	0	0	0	0
Corolla length 4-7 (mm)	0	0	0	0	1	1	0	0	0	0	0

Corolla length 7-11 (mm)	1	1	0	1	1	1	0	1	1	1	1
Calyx Gland density external surface absent	0	0	0	0	0	0	0	0	0	0	0
Calyx Gland density external surface abundant	1	0	1	1	0	0	0	0	0	0	1
Calyx Gland density external surface scarce	0	0	0	0	1	1	1	1	1	1	1
Calyx length in blooming 4-6 (mm)	0	0	1	0	0	0	1	0	0	0	1
Calyx length in blooming 6-8 (mm)	1	1	1	1	0	0	1	1	1	1	1
Calyx teeth length 1-2 (mm)	0	0	0	1	1	1	0	1	1	1	1
Calyx teeth length 2-4 (mm)	1	0	0	1	1	1	0	1	0	1	0
Calyx Trichome density external surface abundant	1	1	1	1	0	0	1	0	1	1	1
Calyx Trichome density external surface scarce	0	1	0	0	0	1	0	0	1	0	1
Carpostegium continuous	1	0	1	1	0	0	1	0	0	0	0
Carpostegium discontinuous	0	1	0	1	1	1	0	1	1	1	1
Abaxial Surface of bracts Trichome density abundant	1	0	0	0	1	1	0	0	1	0	0
Abaxial Surface of bracts Trichome density very scarce to scarce	1	1	1	1	0	0	1	0	1	1	1
Abaxial Surface of bracts glands density absent	0	0	1	1	0	0	1	0	0	0	0
Abaxial Surface of bracts glands density very scarce to scarce	1	0	0	0	1	1	0	0	1	1	1
Abaxial Surface of bracts Trichome length (µm) 2000-3000	0	0	0	0	0	0	0	1	0	0	0
Abaxial Surface of bracts Trichome length (µm) 200-800	1	1	1	1	0	0	0	0	0	0	0
Abaxial Surface of bracts Trichome length (µm) 800-1400	1	0	1	1	1	1	0	0	1	1	0

Table S2. Comparison of *Sideritis carpetana sp. nov*. with other closely-related taxa. Data on *S. glacialis*, *S. pungens* and *S. hyssopifolia* from Obon and Rivera, 1994; Rivera *et al*., 1999; Roselló *et al*., 2000; Ríos *et al*., 2010; Morales, 2016 and studied specimens.

Characters	***Sideritis carpetana* sp. nov.**	*S. glacialis*<br>(Sierra de Gúdar)<br>=*S. glacialis* subsp. *fontqueriana*<br>=*S. fernandez-casasii*	*S. pungens*<br>(Sierra de Javalambre)<br>= *S. pungens* subsp. *javalambrensis*	*Sideritis hyssopifolia* subsp. *nocedoi* (Cantabrian Range)
Growth	decumbent to erect	procumbent	erect	procumbent to erect
Heigth (cm)	10.9-14.7	up to 25	up to 22	up to 45
**Non woody branches**				
Arrangement of hairs	holotrichous	holotrichous	goniotrichous to holotrichous	goniotrichous to holotrichous
Length of hairs (µm)	700-1280	1000-1500	500-1300	700-1000
N. cells of trichomes	3-4	1-3	2-3	2-3
Trichome cell type	cylindrical	band-shaped	cylindrical	cylindrical
Apical trichome cell type	band shaped	band-shaped	conical	conical
**Lower leaves**				
Length (mm)	8-12.5	4-9	10-28	7-17
Width (mm)	2.5-3.5	1-2	2-4	2-4
Shape	linear-oblanceolate to oblanceolate	linear-spatulate	linear to oblanceolate	lanceolate-spatulate
Type of apex	acute to apiculate	acute	apiculate	obtuse
Density of trichomes	scarce	scarce to abundant	scarce to very scarce	very scarce to scarce
Length of trichomes (µm)	700-1260	800-1200	700-1000	200-900
**Inflorescence**				
Length (cm)	2-3	1.5-3	1-6	1-2
Number of verticillasters	5-6	1-7(8)	(1)4-6	2-5
Length trichome axis (µm)	840-1420	1000	1000-2000	800-1200
Number cells trichome axis	3-4	1-2	2-3	2-3
**Lower verticillaster**				
Direction of bracts	erect-patent to	erect-patent	patent	erect-patent
bract shape	ovate or trullate	trullate	ovate	ovate or trullate
bract length (mm)	6.5-9	5-7	8-12	5-8
bract width (mm)	4.8-7.5	6-7	10-12	4-6
Number teeth of semibracts	7-10	9-10(14)	14-16	0-3
**Central verticillaster**				
bract shape	ovate or trullate	trullate	ovate or trullate	erect-patent, ovate
bract length (mm)	6-8	5-6	6-8	5-8
bract width (mm)	6-9.5	5-6	10-13	5-7
semibracts teeth number	6-7	9-10(14)	8-14	0-3
**Abaxial surface of bracts**				
Gland density	very scarce	absent	scarce to very scarce	absent
Trichome density	scarce to abundant	abundant	scarce to very scarce	scarce to very scarce
Trichome length (µm)	800-1300	800-1200	800-1200	200-800
Trichome cell type	cylindrical	cylindrical	cylindrical	cylindrical

Apical cell trichome type	cylindrical/band shaped	conical	conical	conical
Number flowers verticillaster	5-7	1-5	3-10	6
**Calyx**				
Length in blooming (mm)	6-8.3	5-6(8)	7-8	5-6
Length teeth	2.5-3.5	1.5-2.5	1-3	1-2
Length spines	1-1.5	0.5	1	1
Gland density external surface	abundant	scarce or abundant	scarce	scarce
Trichome density external surface	abundant	abundant	scarce to very scarce	scarce to abundant
Trichome length external	760-1420	1000	900-1100	1000-1500
**Corolla**				
length (mm)	8-10	8-10	7-9	5-7
division	1/2-1/4	1/3	1/3	1/3
incision upper lip	entire or slightly emarginate	emarginate	emarginated or notched	entire or emarginate
